# USING SOCIAL MEDIA TO UNDERSTAND FAMILY CAREGIVER PERSPECTIVES ON ASSISTIVE TECHNOLOGY FOR PERSONS WITH ALS

**DOI:** 10.1093/geroni/igac059.2944

**Published:** 2022-12-20

**Authors:** Yong K Choi, Salhah Alomairi

**Affiliations:** University of California Davis School of Medicine, Sacramento, California, United States; University of California Davis School of Medicine, Sacramento, California, United States

## Abstract

ALS Forum (alsforums.com) is an online support group for those who have motor neuron disease or amyotrophic lateral sclerosis (ALS). It provides information, advice, connection, care, and assistance to persons living with ALS and their family caregivers. This study aimed to analyze the online discussion posts by caregivers of persons with ALS and assess their perceptions, experiences, and challenges of using assistive technologies (AT). Posts were extracted from the ALS caregiver discussion forum via web scraping using Python in March 2022. The extracted posts are dated between June 2005 and January 2022. The total number of posts and replies generated during this period was 52,292 by 2,397 unique users. Additional searches were conducted using assistive technology-related keywords to create the text dataset. A total of 263 posts by 137 unique users were identified. After the initial review, 186 posts were included in the final analysis. Thematic analysis revealed six major themes: (1) Uses of AT (automation/control, communication, mobility); 2) Barriers and Facilitators (cost, effort to learn new technology, rejection, interoperability, customer service); 3) Usability (hardware and software-related); 4) Sharing Experience on AT; 5) Giving Advice; 6) Seeking Information on AT. The findings are consistent with previous literature that online health discussion forums provide access to rich data that contains lived experiences of patients and caregivers. The findings may inform the design of future assistive technology that incorporates the patients’ and caregivers’ perspectives to increase the adoption and long-term sustainability of the technology for people living with ALS and their caregivers.

